# Dendritic cells transduced with TIPE-2 recombinant adenovirus induces T cells suppression

**DOI:** 10.1186/s12950-021-00274-8

**Published:** 2021-02-10

**Authors:** Shudong Liu, Jie Wang, Wenyan Li, Hui Shi, Changlong Zhou, Ge Tang, Jiangwei Zhang, Zhao Yang

**Affiliations:** 1 Department of Neurology and Chongqing Key Laboratory of Cerebrovascular Disease, Yongchuan Hospital, Chongqing Medical University, Chongqing, 402160 China; 2 Department of Neurology, Chongqing traditional Chinese medicine hospital, Chongqing, 400021 China; 3 Department of Neurosurgery, Yongchuan Hospital, Chongqing Medical University, Chongqing, 402160 China

**Keywords:** Dendritic cells, TIPE-2, Adenovirus, T cells suppression

## Abstract

**Introduction:**

TIPE-2 has been identified as a negative regulator of both innate and adaptive immunity and is involved in several inflammatory diseases. However, the role of immune suppression of dendritic cells (DCs) transduced with TIPE-2 has not been well studied.

**Methods:**

In this study, DCs were transduced with TIPE-2 recombinant adenovirus, and then were cocultured with allogeneic CD4+ or CD8 + T cells. The proliferation, cytokine production and activation marker levels of CD4+ or CD8 + T cell were detected.

**Results:**

The data demonstrated that T cell proliferation, cytokine production and activation marker levels were attenuated after treated with TIPE-2 transduced DCs.

**Conclusions:**

These results suggested that TIPE-2 transduced DCs are capable of inducing allogeneic CD4+ or CD8 + T cell immune suppression, which provide a promising way for the therapeutical strategies of transplantation or autoimmune diseases.

## Introduction

Immune suppression is the ultimate strategy of transplantation or autoimmune diseases [1–3]. Among enormous methods, induction of T cell suppression appears to be most ideal way because T cells are a significant factor affecting the outcome of autoimmune disease [4–6]. T cell responses are manipulated by a complex network of activating and attenuator molecules. Besides peptides/MHC (major histocompatibility complex) complex, additional signals from costimulatory molecules are important for T cell activation [7–9].

TIPE-2, the tumor necrosis factor (TNF)-induced protein 8-like 2, makes a crucial effect in keeping immune homeostasis by negatively regulating T cell receptor and Toll-like receptor (TLR) signaling [10, 11]. TIPE-2 is preferentially expressed in lymphoid tissues, macrophages and lymphocytes [12–14]. TIPE-2 was identified to contribute to inflammatory and infectious diseases, such as hepatitis B, systemic lupus erythematosus, and asthma [15–17] . Related evidence suggests that TIPE-2 can negative regulate innate and adaptive immune response [18–20].

Dendritic cells (DC) are the most potent professional antigen- presenting cells (APC) and have the most powerful antigen-presenting capacity [21]. Recent studies demonstrate that DCs contributed to the maintenance of immunological self-tolerance [22]. In vivo transfer of Ag-loaded DCs with a tolerogenic character acted as a promising way to attenuate the immune response [23]. DCs transduced with recombinant replication-defective adenoviral (Ad) vectors have been improved to be an efficient alternative for gene transfer study [24].

The immune suppression capability of DCs transduced with TIPE2 recombinant adenovirus has not been reported. In this experiment, DCs were transduced with recombinant adenovirus encoding TIPE2, and then cocultured with allogeneic CD4+ or CD8 + T cells. The potential of TIPE2 transduced DCs to induce T cell suppression was detected.

## Materials and methods

### Construction of recombinant adenovirus encoding TIPE-2

The recombinant adenovirus vector encoding TIPE-2 was constructed using the Adeno-XTM Expression System (Clontech, Palo Alto, CA, USA) according to the manufacturer’s instructions. Briefly, the TIPE2 cDNA was cloned into the shuttle vector pDC315 and sequenced. Amplification conditions were: 95 °C for 3 min and then 95 °C for 15 s, 55 °C for 15 s and 72 °C for 30 s for 40 cycles. Primers used for this study were synthesized by Invitrogen Corporation and shown as follows:

5`-TCAGAAACATCCAAGGCCAGAC-3`(sense) and 5`-CGG ACC GA CCAGCCATTTTAC-3` (antisense) for TIPE-2. The desired replication- deficient adenovirus containing the full-length cDNA of TIPE-2 was generated by homologous recombination through cotransfection of plasmids pDC315-TIPE-2 and pBHG1oXE1, 3Cre in HEK 293 cells using the DOTAP liposome reagent (Roche, Germany). After several rounds of plaque purification, the adenovirus containing the TIPE-2 gene was amplified and purified from cell lysates by banding twice in CsCl density gradients. Viral products were desalted and stored at − 80 °C in phosphate-buffered saline (PBS) containing 10% glycerol (v/v). The infectious titer was determined by a standard plaque assay. A second recombinant El-, E3-deleted adenovirus carrying the LacZ protein under the control of CMV promoter (rAd-LacZ) was used as a control vector for DC transduction.

### Dendritic cell generation

Briefly, 1 × 10^7^ PBMCs were isolated from healthy donors (200 ml whole blood) by Ficoll-Hypaque density gradient centrifugation and then seeded into culture flasks in RPMI-1640 medium supplemented with penicillin (100 U/ml), streptomycin (100 μg/ml), and 10% FBS. The adherent cells were cultured for 5 days in RPMI-1640 containing 1000 U/ml of granulocyte-macrophage colony-stimulating factor (R&D Systems, Inc., Minneapolis, MN) and interleukin-4 (IL-4; R&D Systems, Inc.), and were the cultured for an additional 2 days in the presence of 1000 U/ml of tumor necrosis factor α (R&D Systems, Inc.) to induce final maturation. After 7 days of culture, the mature DCs were harvested and analyzed for DC typical phenotypes by FACS analysis.

### FACS analysis of DC phenotypes

DCs were collected and resuspended in cold FACS buffer (phosphate- buffered saline with 0.2% BSA and 0.09% sodiumazide). Cells were immunostained with FITC-conjugated mouse anti-human CD11c, CD83, and CD86 antibodies (eBioscience, USA). The appropriate FITC isotype antibody (eBioscience, USA) was used as a control. A total of 1 × 10^6^ cells were incubated for 4 h at 4 °C with antibodies. The cells were washed once with FACS buffer and then resuspended and phenotyped on a FACScan (Becton-Dickinson, USA).

### Adenovirus-mediated gene transfer

In a 24-well plate, DCs were seeded at a density of 5 × 10^4^ cells/well and incubated for approximately 24 h until 50–70% confluent in flasks with DMEM supplemented with 10% (v/v) FBS and 1% penicillin- streptomycin at 37 °C in a humidified atmosphere of 5% CO_2_. Virus was added to the wells at an MOI of 200 and DCs were harvested after 24 h of incubation.

### Western blot assay

For Western blot assay, proteins of the cell extracts were separated SDS-PAGE and transferred onto a nitrocellulose membrane. The membrane was incubated with 5% non-fat milk in PBS and then with anti-TIPE-2 antibody (Santa Cruz Biotechnology, Santa Cruz, CA, USA) for 2 h at room temperature. After washing, the membranes were incubated with an alkaline phosphatase-conjugated goat antimouse IgG antibody (Amersham Biosciences, Little Chalfont, UK) for 1 h at room.

temperature. Immunoreactive bands were detected using the ECL Western blot analysis system (Amersham Biosciences).

### Coculture of DCs and T cells by one-way MLRs assay

DCs were prepared using the method described above. Single cell suspensions were treated with mitomycin C (50 μg/ml; Sigma-Aldrich) for 20 min at room temperature and were then washed twice with RPMI 1640. These cells were used as the stimulator cells in the assay. Responder CD4 + or CD8 + T cells were harvested by negative isolation using magnetic beads according to the manufacturer’s protocol (Dynal T Cell Negative Isolation kit; Invitrogen Life Technologies). The purity of the CD4 + or CD8 + T cells was determined by FACS (BD Biosciences); only CD4 + or CD8 + T cell populations with > 95% purity was used in this study. Responder (2 × 10^5^) and stimulator (4 × 10^5^) cells were added to round-bottom 96-well plates to a final volume of 200 μl RPMI1640 with 10% FCS. Each experiment was performed in triplicate.

### Proliferation assays

Cells activated in an MLR were allowed to incubate for up to 3 days before harvesting. [3H]Thymidine (0.5 μCi/well; MPBiomedicals) was added 24 h before harvesting (SkatronInstruments) using Type A filter mats (Perkin-Elmer Life and Analytical Sciences) and a beta plate scintillation mixture (Perkin-Elmer). Disintegrations per minute were determined using a liquid scintillation counter (1205 Betaplate; Perkin-Elmer).

### ELISA assay for quantitating cytokine production

X-ray-irradiated DC (2 × 10^4^) and the T cells (1 × 10^5^) were seeded into wells of 96-well flat-bottom culture plates in RPMI 1640 10% fetal bovine serum (Invitrogen Life Technologies) for 3 days. Supernatants were removed and further analyzed for cytokine production (IL-2 and IFN-γ) with ELISA. Plates were read at 405 nm using a Labsystems Multiskan enzyme-linked immunosorbent assay reader.

### Flow cytometry for detecting activation marker expression

FCM assay was used to characterize activation marker expression of T cells. CD4 + or CD8 + T cells were cocultured with DCs for 3 days. The.

cells were washed with PBS and then stained with anti-CD3-PE,

anti-CD25-FITC and anti-CD69-APC at 4 °C for 1 h, and then washed by PBS. CD25 and CD69 activation markers were measured using a FCM Calibur flow cytometer (BD company). Data were analyzed with CellQuest software.

### Statistical analysis

The statistical significance of differential findings between experimental groups and controls was determined by Student’s t-test and considered significant if two-tailed *P*-values were < 0.05. The *sample size is n = 10 in each group.*

## Results

### FACS analysis of DC phenotypes

**Fig. 1:**
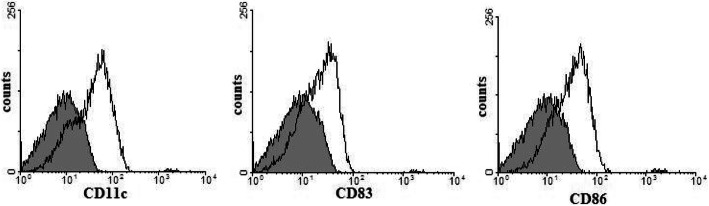
FACS analysis of DC phenotypes Human peripheral blood mononuclear cells (PBMCs) were isolated from the whole blood of normal donors. PBMC monocytes were purified in complete culture medium for 90 min. The remaining adherent cells were induced by 800 U/ ml recombinant GM-CSF and 1000 U/ ml IL-4 for 5 days. Cells were cultured for 2 days in the presence of 1000 U/ ml TNF-α to induce final maturation. The cells were immunostained with FITC and anti-CD11c, CD83 and CD86 antibodies, and the phenotypes were analyzed by flow cytometry. These mature dendritic cells express high levels of CD11c, CD83 and CD86. Three repeated experiments showed similar results

On day 7 of cell culture, DCs were harvested from monocytes cultured in medium containing GM-CSF, IL-4, and TNF-α. To analyze cell phenotype, mature DCs were detected by FACS. The data demonstrated that these mature DCs expressed high levels of CDllc (83.5%), CD83 (85.1%) and CD86 (81.7%) (Fig. 1).

### TIPE-2 gene transduction

**Fig. 2:**
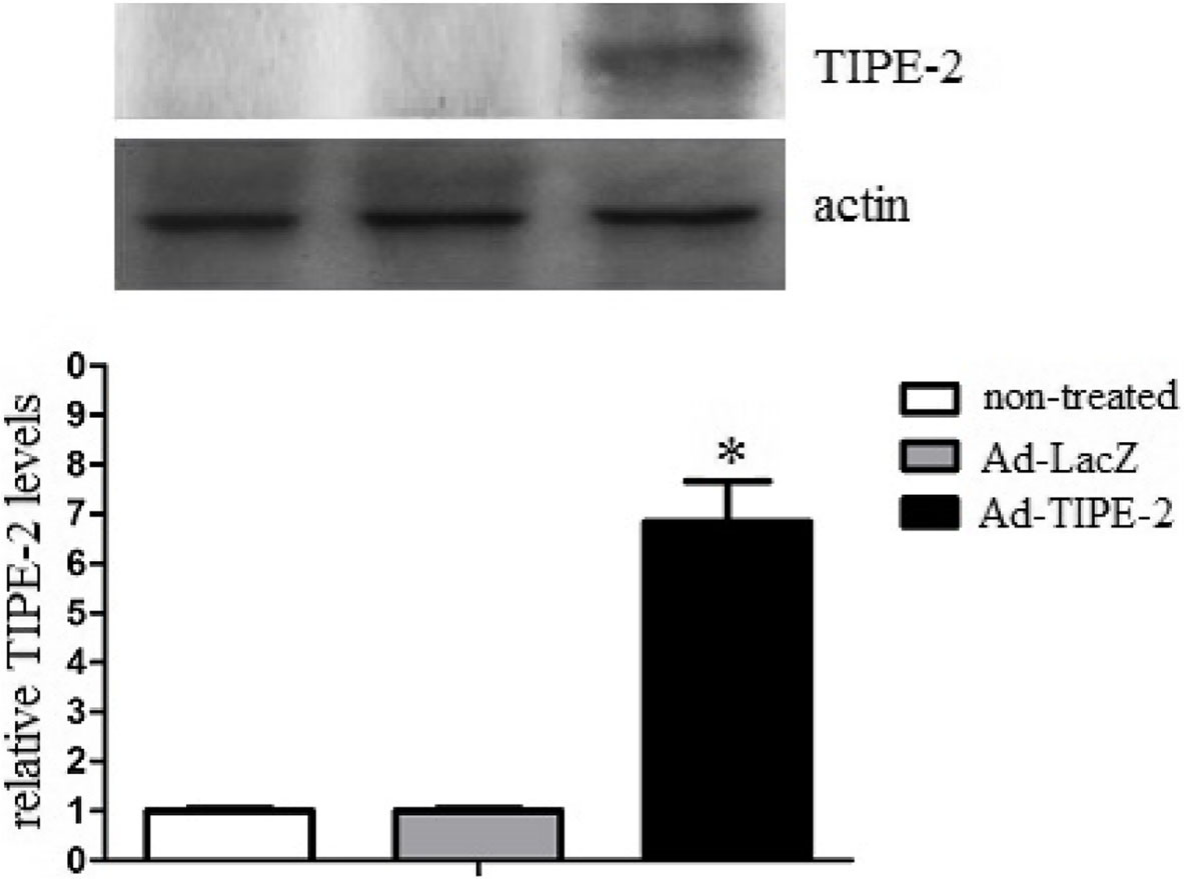
Western blot analysis of TIPE-2 protein. DCs were transduced with Ad-TIPE-2 or Ad-LacZ at an MOI of 200 for 24 h. The cell lysates were collected and further analyzed for TIPE-2 levels. TIPE-2 protein was detected after Ad-TIPE-2 transduction. However, TIPE-2 could be hardly detected after Ad-LacZ transduction or in non-treated DCs. Lane1: non-treated DCs; lane2: DCs tranduced with Ad-LacZ; lane3: DCs tranduced with Ad-TIPE-2. Three repeated experiments showed similar results

In accordance with protocols mentioned above, DCs were transduced with Ad-TIPE-2 or Ad-LacZ at MOI 100 for 24 h. The transfection efficiency was analyzed by FACS. The FACS data demonstrated that TIPE-2 positive DC ratio is (73.6%) after Ad-TIPE-2 transfection (data not shown). In addition, TIPE-2 protein levels were analyzed by Western blot assay. The results demonstrated that the expression of the TIPE-2 protein was detected after Ad-TIPE-2 transduction. However, the expression of TIPE-2 protein after Ad-LacZ transduction and non-treated DCs could hardly been detected (Fig. 2).

### Ad-TIPE-2-transduced DCs attenuate CD4 + and CD8 + T cell proliferation

**Fig. 3:**
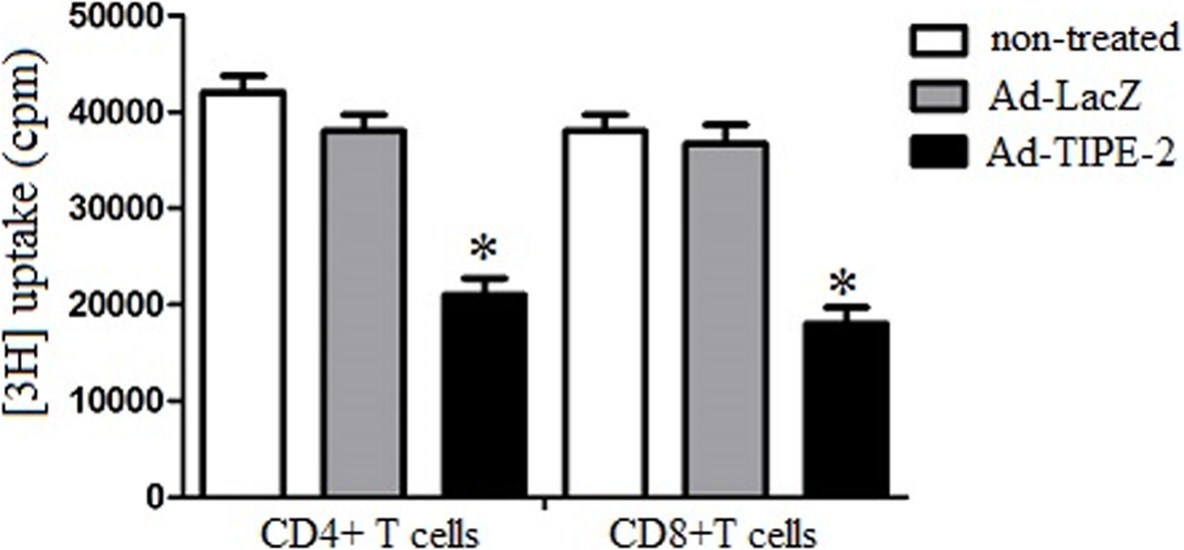
CD4+ and CD8+ T cell proliferation assay. Transduced DCs were treated with mitomycin C (50 μg/ml) for 20 min at room temperature to act as stimulator cells. Allogeneic responder CD4 + or CD8 + T cells were collected by CD4 or CD8 negative isolation with magnetic beads. CD4+ or CD8+ T cells (2 × 10^5^) were treated with transduced DCs (4 × 10^5^) for 3 days. [3H] Thymidine (0.5 μCi/well) was added 24 h before harvesting using TypeA filtermats and a beta plates cintillation mixture. Disintegrations per minute were determined using a liquid scintillation counter (1205 Betaplate; Perkin-Elmer). Histogram numbers represent the mean ± S.E.M. for each experiment. Three repeated experiments showed similar results

To explore the immune suppression of TIPE-2 on T cell proliferation, we observed maximum proliferation of cells on day 3 of culture in the one-way MLR assay. Briefly, Ad-TIPE-2-transduced DCs were cocultured with CD4 + or CD8 + T cells for 3 days using the methods described above. [3H] Thymidine (0.5 μCi/well; MP Biomedicals) was added to detect and quantify proliferation. The results demonstrated that Ad-TIPE-2-transduced DCs decreased CD4+ or CD8+ T cell proliferation (2.13 × 10^4^ cpm or 1.87 × 10^4^ cpm) compared to Ad-LacZ (3.81 × 10^4^ cpm or 3.69 × 10^4^ cpm) and untreated DC controls (4.15 × 10^4^ cpm or 3.83 × 10^4^ cpm) (*P* < 0.05) (Fig. 3). These results suggested that Ad-TIPE-2 -transduced DCs could attenuate CD4 + and CD8 + T cell proliferation.

### Ad-TIPE-2-transduced DCs attenuate cytokine release of CD4 + and CD8 + T cells

**Fig. 4:**
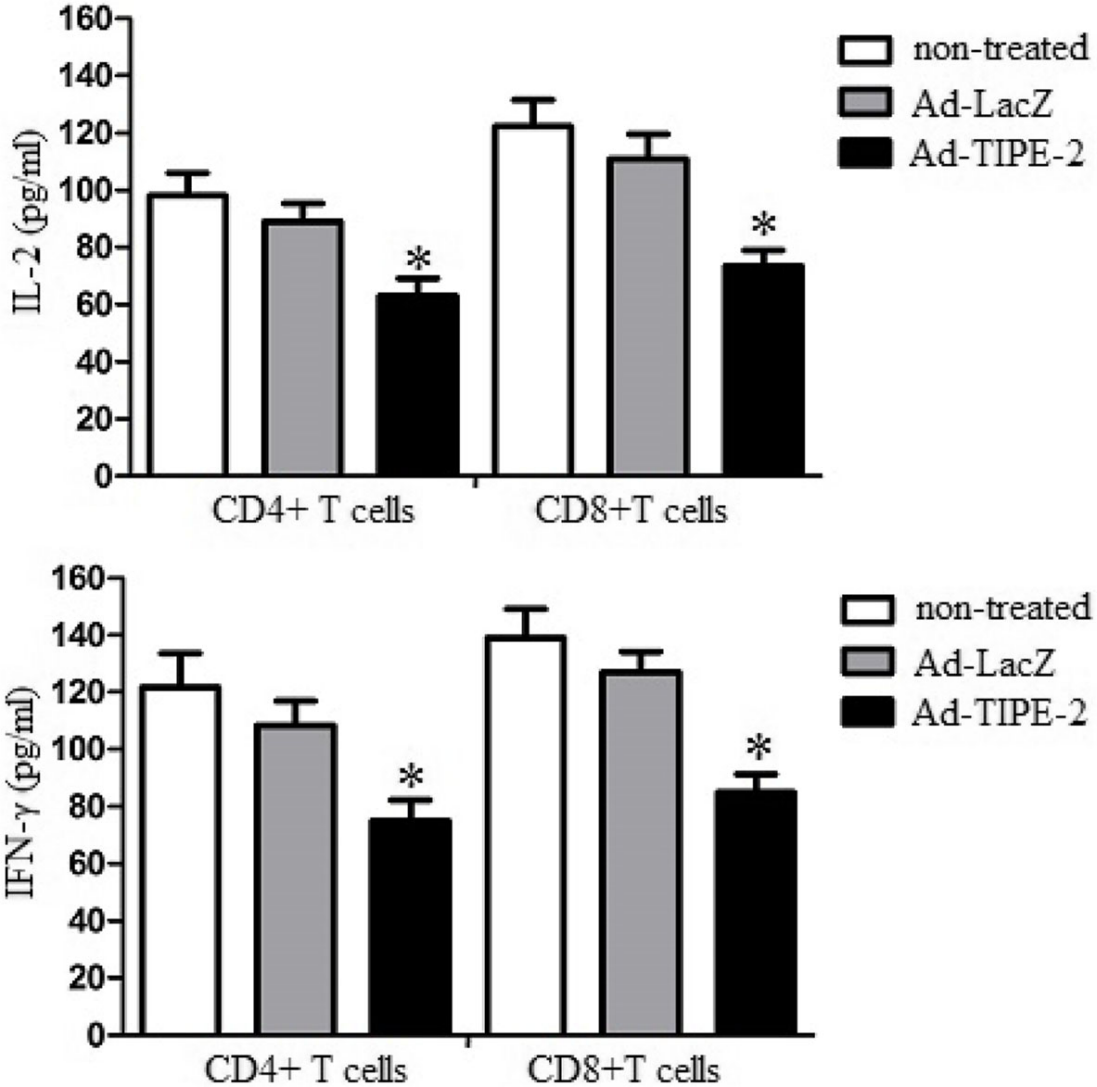
CD4 + and CD8 + T cell cytokine levels. CD4+ or CD8+ T cells (2 × 10^5^) were treated with transduced DCs (4 × 10^5^) for 3 days. Supernatants were removed and further analyzed for cytokine production (IL-2 and IFN-γ) with ELISA. Plates were read at 405 nm using a Labsystems Multiskan enzyme-linked immunosorbent assay reader. Histogram numbers represent the mean ± S.E.M. for each experiment. Three repeated experiments showed similar results

To explore the immune suppression of TIPE-2 on T cell activation, we analyzed CD4 + or CD8 + T cell cytokine production by ELISA assay. Briefly, Ad-TIPE-2-transduced DCs were cocultured with CD4 + or CD8 + T cells for 3 days as described above. The supernatant was harvested and analyzed by ELISA according to standard methods. The results demonstrated that Ad-TIPE-2-transduced DCs decreased IL-2 (61.4 pg/ml or 71.8 pg/ml) and IFN-γ (76.3 pg/ml or 81.5 pg/ml) production of CD4 + or CD8 + T cells compared to Ad-LacZ-transduced control cells (IL-2: 83.4 pg/ml or 116.2 pg/ml; IFN-γ:114.2 pg/ml or 123.3 pg/ml) (*P* < 0.05) (Fig. 4). These results suggested that Ad-TIPE-2-transduced DCs could attenuate CD4 + and CD8 + T cell cytokine production.

### Ad-TIPE-2-transduced DCs attenuate CD4+ and CD8 + T cells activation markers levels

**Fig. 5:**
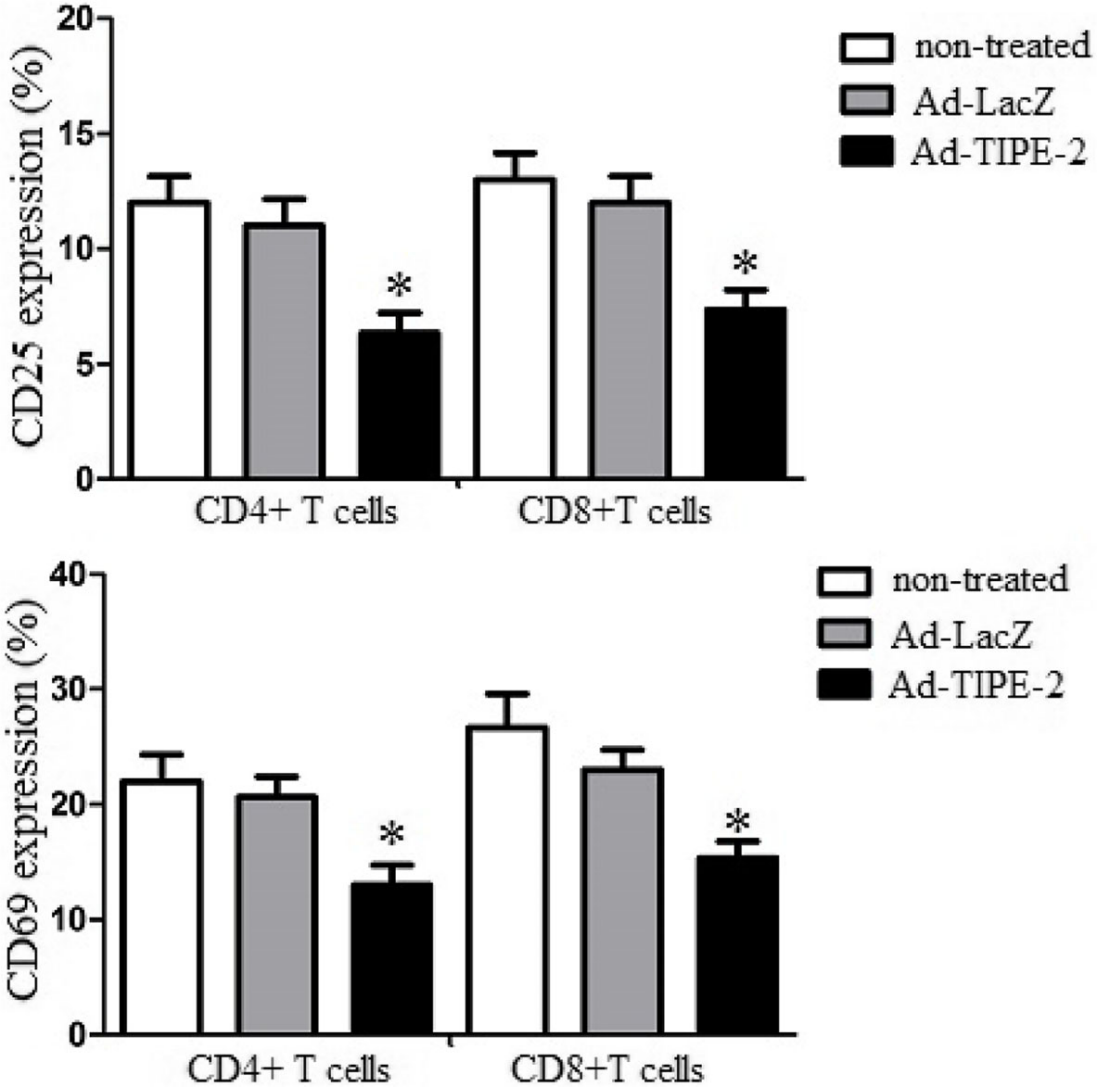
CD4 + and CD8 + T cells activation marker levels. FCM assay was used to characterize activation marker expression of T cells. CD4 + or CD8 + T cells were cocultured with DCs for 3 days. The cells were washed with PBS and then stained with anti-CD3-PE, anti-CD25-FITC and anti-CD69-APC at 4 °C for 1 h, and then washed by PBS. CD25 and CD69 activation markers were measured using a FCM Calibur flow cytometer (BD company). Histogram numbers represent the mean ± S.E.M. for each experiment. Three repeated experiments showed similar results

To explore the immune suppression of TIPE-2 on T cell activation, we analyzed CD4+ or CD8+ T cells activation marker levels by flow cytometry. Briefly, Ad-TIPE-2-transduced DCs were cocultured with CD4+ or CD8 + T cells for 3 days using the methods mentioned above. The results demonstrated that Ad-TIPE-2-transduced DCs decreased activation markers CD25 (6.4% or 7.6%) and CD69 (12.3% or 15.7%) levels of CD4+ or CD8 + T cells compared to Ad-LacZ-transduced control cells (CD25: 11.4% or 13.8%; CD69: 20.6% or 22.8%) (*P* < 0.05) (Fig. 5). These results suggested that TIPE-2 could inhibit CD4 + and CD8 + T cells activation marker levels.

## Discussion

In current experiment, we provided the evidence that Ad-TIPE-2-transduced DCs induced T cell immune suppression, leading to T cell proliferation, cytokine production and activation marker level inhibition. These findings have not been reported previously.

TIPE-2 is a member of the tumor necrosis factor-α-induced protein 8 family which performs diverse functions, including the negative regulation of innate and adaptive immunity, transcription factor AP-1 and nuclear factor (NF)-κB activation and tumor suppression [25–27]. Consequently, TIPE-2-deficient mice have been identified to exhibit increased M1 inflammation and resistance to M2 polarization, and the deficiency may attenuate systemic lupus erythematosus autoimmunity via macrophage polarization [28].

Dendritic cells are the most effective APCs, which are responsible for initiating the immune response of naive T cells, and participating in maintaining immune self tolerance, promoting T cells with regulatory function or inducing T cell anergy [29–31]. In vivo transfer of Ag-loaded DC with a tolerogenic character is considered to be a promising treatment for Ag specific negative regulation of immune response [32–34]. Genetic modification may represent a reliable way to manipulate DC characteristics. Generation of tolerogenic DC by forced expression of Fas ligand, indoleamine 2,3-dioxygemase, IL-10, or CTLA4Ig with gene transfer assays has also been identified [35–37]. However, the ability of TIPE-2 recombinant adenovirus to induce T cell inhibition by dendritic cells has not been studied. Therefore, we constructed the recombinant adenovirus of TIPE-2 and used it to transduce DCs to observe its effect on T cells.

Firstly, we used [3H] Thymidine assay to investigate the inhibitory effect of DCS on the proliferation of allogeneic CD4 + and CD8 + T cells. The results indicated that TIPE-2 inhibited allogeneic CD4+ and CD8 + T cell proliferation. Secondly, we used ELISA assay to analyze one-way MLR test. The results indicated that TIPE-2 inhibited allogeneic CD4+ and CD8 + T cell cytokine production. Lastly, we used flow cytometry to assess the activation marker. The results indicated that TIPE-2 inhibited allogeneic CD4+ and CD8 + T cell activation.

## Conclusions

In conclusion, our studies suggested that TIPE-2 had the capability to induce T cell immune suppression. In addition, Ad-TIPE-2 transduced DCs represented an ideal target for transplantation or autoimmune diseases.

## Data Availability

The datasets used and analyzed during the current study are available from the corresponding author on reasonable request.

## References

[1] Allison TL (2016). Immunosuppressive Therapy in Transplantation. Nurs Clin North Am..

[2] Lim MA, Kohli J, Bloom RD (2017). Immunosuppression for kidney transplantation: Where are we now and where are we going?. Transplant Rev (Orlando)..

[3] Flowers ME, Martin PJ. How We Treat Chronic Graft-Vsersus-Host Disease. Blood. 2015;125(4):606–15. 10.1182/blood-2014-08-551994 PMC4304105 25398933

[4] Ligocki AJ, Niederkorn JY (2015). Advances on Non-CD4 + Foxp3+ T Regulatory Cells: CD8+, Type 1, and Double Negative T Regulatory Cells in Organ Transplantation. Transplantation..

[5] Sinha S, Subramanian S, Proctor TM, Kaler LJ, Grafe M, Dahan R, Huan J, Vandenbark AA, Burrows GG, Offner H (2007). A promising therapeutic approach for multiple sclerosis: recombinant T-cell receptor ligands modulate experimental autoimmune encephalomyelitis by reducing interleukin-17 production and inhibiting migration of encephalitogenic cells into the CNS. J Neurosci..

[6] Astorri E, Nerviani A, Bombardieri M, Des PCCP (2015). Towards a stratified targeted approach with biologic treatments in rheumatoid arthritis: role of synovial pathobiology. Curr Pharm Des..

[7] Meryk A, Pangrazzi L, Hagen M, Hatzmann F, Jenewein B, Jakic B, Hermann-Kleiter N, Baier G, Jylhävä J, Hurme M, Trieb K, Grubeck-Loebenstein B (2019). Fcμ receptor as a Costimulatory Molecule for T Cells. Cell Rep..

[8] Ogawa S, Abe R (2019). Signal Transduction Via Co-stimulatory and Co-inhibitory Receptors. Adv Exp Med Biol..

[9] Rhodes KR, Green JJ (2018). Nanoscale artificial antigen presenting cells for cancer immunotherapy. J. Mol Immunol..

[10] Sun H, Gong S, Carmody RJ, Hilliard A, Li L (2008). TIPE2, a negative regulator of innate and adaptive immunity that maintains immune homeostasis. Cell.

[11] Freundt EC, Bidere N, Lenardo MJ (2008). A different TIPE of immune homeostasis. Cell.

[12] Zhang G, Hao C, Lou Y, Xi W, Wang X (2010). Tissue-specific expression of TIPE2 provides insights into its function. Mol Immunol.

[13] Laliberte B, Wilson AM, Nafisi H, Mao H, Zhou YY (2010). TNFAIP8: a new effector for Galpha(i) coupling to reduce cell death and induce cell transformation. J Cell Physiol.

[14] Li D, Song L, Fan Y, Li X, Li Y (2009). Down-regulation of TIPE2 mRNA expression in peripheral blood mononuclear cells from patients with systemic lupus erythematosus. Clin Immunol.

[15] Lou Y, Liu S (2011). The TIPE (TNFAIP8) family in inflammation, immunity. and cancer. Mol Immunol.

[16] Xi W, Hu Y, Liu Y, Zhang J, Wang L (2011). Roles of TIPE2 in hepatitis B virus-induced hepatic inflammation in humans and mice. Mol Immunol.

[17] Zhang L, Shi Y, Wang Y, Zhu F, Wang Q (2011). The unique expression profile of human TIPE2 suggests new functions beyond its role in immune regulation. Mol Immunol.

[18] Zhang Y, Wei X, Liu L, Liu S, Wang Z (2012). TIPE2, a novel regulator of immunity, protects against experimental stroke. J Biol Chem.

[19] Gus-Brautbar Y, Johnson D, Zhang L, Sun H, Wang P (2012). The anti-inflammatory TIPE2 is an attenuateor of the oncogenic Ras. Mol Cell.

[20] Luan YY, Yao YM, Zhang L, Dong N, Zhang QH (2011). Expression of tumor necrosis factor-alpha induced protein 8 like-2 contributes to the immunosuppressive property of CD4(+)CD25(+) regulatory T cells in mice. Mol Immunol.

[21] Lancaster JN, Thyagarajan HM, Srinivasan J, Li Y, Hu Z, Ehrlich LIR (2019). Live-cell imaging reveals the relative contributions of antigen-presenting cell subsets to thymic central tolerance. Nat Commun..

[22] Xiao Y, Zou Q, Xie X, Liu T, Li HS, Jie Z, Jin J, Hu H, Manyam G, Zhang L, Cheng X, Wang H, Marie I, Levy DE, Watowich SS, Sun SC (2017). The kinase TBK1 functions in dendritic cells to regulate T cell homeostasis, autoimmunity, and antitumor immunity. J Exp Med..

[23] Flórez-Grau G, Zubizarreta I, Cabezón R, Villoslada P, Benitez-Ribas D (2018). Tolerogenic Dendritic Cells as a Promising Antigen-Specific Therapy in the Treatment of Multiple Sclerosis and Neuromyelitis Optica From Preclinical to Clinical Trials. Front Immunol.

[24] Senesac J, Gabrilovich D, Pirruccello S, Talmadge JE (2014). Dendritic cells transfected with adenoviral vectors as vaccines. Methods Mol Biol..

[25] Zhang G, Zhang W, Lou Y, Xi W, Cui J (2013). TIPE2 deficiency accelerates neointima formation by downregulating smooth muscle cell differentiation. Cell Cycle.

[26] Jia L, Gui B, Tian P, Yao G, Fu R (2013). TIPE2, a novel biomarker for clinical chronic kidney allograft rejection. Artif Organs.

[27] Wang Z, Fayngerts S, Wang P, Sun H, Johnson DS (2012). TIPE2 protein serves as a negative regulator of phagocytosis and oxidative burst during infection. Proc Natl Acad Sci U S A.

[28] Jiang Y, Li Q, Zhang Y, Gao Y, Jiang L (2018). TIPE2 governs macrophage polarization via negative regulation of mTORC1. Mol Med Rep.

[29] Schülke S (2018). Induction of Interleukin-10 Producing Dendritic Cells As a Tool to Suppress Allergen-Specific T Helper 2 Responses. Front Immunol..

[30] Yu H, Tian Y, Wang Y, Mineishi S, Zhang Y (2019). Dendritic Cell Regulation of Graft-Vs.-Host Disease: Immunostimulation and Tolerance. Front Immunol..

[31] Wu J, Zhang H, Zheng Y, Jin X, Liu M, Li S, Zhao Q, Liu X, Wang Y, Shi M, Zhang S, Tian J, Sun Y, Zhang M, Yu B (2018). The Long Noncoding RNA MALAT1 Induces Tolerogenic Dendritic Cells and Regulatory T Cells via miR155/Dendritic Cell-Specific Intercellular Adhesion Molecule-3 Grabbing Nonintegrin/IL10 Axis. Front Immunol..

[32] Sato K, Yamashita N, Baba M, Matsuyama T. Modified myeloid dendritic cells act as regulatory dendritic cells to induce anergic and regulatory T cells. Blood 2003; 101(May (9)):3581–3589. 10.1182/blood-2002-09-2712 12511411

[33] Sato K, Yamashita N, Yamashita N, Baba M, Matsuyama T. Regulatory dendritic cells protect mice from murine acute graft-versus-host disease and leukemia relapse. Immunity 2003; 18(March (3)): 367–379. 10.1016/s1074-7613(03)00055-4 12648454

[34] Steinbrink K, Wolfl M, Jonuleit H, Knop J, Enk AH. Induction of tolerance by IL-10-treated dendritic cells. J Immunol 1997; 159(November (10)): 4772–4780. 9366401

[35] Terness P, Bauer TM, Rose L, Dufter C, Watzlik A, Simon H, et al. Inhibition of allogeneic T cell proliferation by indoleamine 2,3-dioxygenase-expressing dendritic cells: mediation of suppression by tryptophan metabolites. J Exp Med 2002; 196(August (4)):447–457. 10.1084/jem.20020052 PMC2196057 12186837

[36] Min WP, Gorczynski R, Huang XY, Kushida M, Kim P, Obataki M, et al. Dendritic cells genetically engineered to express Fas ligand induce donor-specific hyporesponsiveness and prolong allograft survival. J Immunol 2000; 164(January (1)): 161–167. 10.4049/jimmunol.164.1.161 10605007

[37] Lu L, Gambotto A, Lee WC, Qian S, Bonham CA, Robbins PD, et al. Adenovi-ral delivery of CTLA4Ig into myeloid dendritic cells promotes their in vitro tolerogenicity and survival in allogeneic recipients. Gene Ther 1999; 6(April (4)):554–563. 10.1038/sj.gt.3300862 10476215

